# Health literacy of organisations – a cornerstone for fair health outcome

**DOI:** 10.1093/eurpub/ckac130.160

**Published:** 2022-10-25

**Authors:** M Magnusson

**Affiliations:** SV Hospital Group, Angered Hospital, Angered, Sweden

## Abstract

Unhealthy food habits are included in the factors behind several severe health conditions. Their unequal distribution in the population has a complex background. Putting the problem “How can we make our resources more reachable?” instead of “How can we reach these groups?” changes focus from individual to organisational health literacy which opens windows of opportunity. A public health unit with commission to contribute to close health gaps identified a need to systematically develop its own health literacy. Critical examining was conducted by the quan/qual tool Health equlibrium methodology. Reflections on accessability and acceptability of resources offered by the unit were documented and used for methodolocigal development. Aims were to develop professional judgment on how to contribute to fair health outcome and to improve support for healthy habits. Data used were collected 2019-2021.What hinders people from healthier food habits? How can we adjust our practice? Documentation included organised breakfast-talks, food-talk with cultural interpreters, lectures with sports-club health ambassadors, health groups with people of different maternal language, meetings with parents at open pre-school, staff in health promotion commissions and elderly. Problems identified were high costs on healthy food and on travels to vending points, traditional large sugar-intake, marketing of unhealthy food to children, failure to understand information from Swedish Food Agency (except the Keyhole food labelling which was much appreciated). A model for shop-walks with cultural interpreters, more accessible versions of leaflet-materials and dialogue-meetings about food in different settings were developed. Reflections on the unit's communication lead to change of settings for meetings and refined ways to talk about parenthood, women's role and aspects of ethnicity. Systematic self-reflection strenghtens organisational health literacy and may contribute to fair health outcomes

**Key messages:**

- Organisational health literacy need to be developed purposedly.
- Scarcity is a barrier for a healthy diet also in a welfare state like Sweden.

